# Defining the representativeness heuristic in trauma triage: A retrospective observational cohort study

**DOI:** 10.1371/journal.pone.0212201

**Published:** 2019-02-08

**Authors:** Shreyus S. Kulkarni, Barry Dewitt, Baruch Fischhoff, Matthew R. Rosengart, Derek C. Angus, Melissa Saul, Donald M. Yealy, Deepika Mohan

**Affiliations:** 1 Department of Surgery, University of Pittsburgh, Pittsburgh, Pennsylvania, United States of America; 2 Department of Engineering and Public Policy, Carnegie Mellon University, Pittsburgh, Pennsylvania, United States of America; 3 Department of Critical Care Medicine, University of Pittsburgh, Pittsburgh, Pennsylvania, United States of America; 4 Department of Medicine, University of Pittsburgh, Pittsburgh, Pennsylvania, United States of America; 5 Department of Emergency Medicine, University of Pittsburgh, Pittsburgh, Pennsylvania, United States of America; National Yang-Ming University, TAIWAN

## Abstract

**Background:**

Under-triage of severely injured patients presenting to non-trauma centers (failure to transfer to a trauma center) remains problematic despite quality improvement efforts. Insights from the behavioral science literature suggest that physician heuristics (intuitive judgments), and in particular the representativeness heuristic (pattern recognition), may contribute to under-triage. However, little is known about how the representativeness heuristic is instantiated in practice.

**Methods:**

A multi-disciplinary group of experts identified candidate characteristics of “representative” severe trauma cases (e.g., hypotension). We then reviewed the charts of patients with moderate-to-severe injuries who presented to nine non-trauma centers in western Pennsylvania from 2010–2014 to assess the association between the presence of those characteristics and triage decisions. We tested bivariate associations using χ^2^ and Fisher’s Exact method and multivariate associations using random effects logistic regression.

**Results:**

We identified 235,605 injured patients with 3,199 patients (1%) having moderate-to-severe injuries. Patients had a median age of 78 years (SD 20.1) and mean Injury Severity Score of 10.9 (SD 3.3). Only 759 of these patients (24%) were transferred to a trauma center as recommended by the American College of Surgeons clinical practice guidelines. Representative characteristics occurred in 704 patients (22%). The adjusted odds of transfer were higher in the presence of representative characteristics compared to when they were absent (aOR 1.7, 95% CI: 1.4–2.0, p < 0.001).

**Conclusions:**

Most moderate-to-severely injured patients present without the characteristics representative of severe trauma. Presence of these characteristics is associated with appropriate transfer, suggesting that modifying physicians’ heuristics in trauma may improve triage patterns.

## Introduction

The American College of Surgeons’ Committee on Trauma (ASC-COT) and the National Academy of Medicine advocate regionalized trauma services to optimize care.[1, 2] Moderate-to-severely-injured patients reap benefits when treated at trauma centers including improved survival, higher rate of discharge of home, and better long-term functional outcomes.[3–5] Despite numerous quality improvement efforts by major stakeholders, up to 70% of these patients do not receive treatment at trauma centers (*under-triage)*.[6] Various institutional and patient-level factors have been shown to contribute to under-triage, but physician judgment may also play a role.[7, 8]

Physicians make diagnostic and management decisions in complex and uncertain circumstances.[9] They rely on heuristic cognitive processes, using mental shortcuts involving simplifying assumptions and pattern recognition.[10] When heuristics are well-calibrated (i.e., aligned with clinical practice guidelines), they guide efficient and reasonable decision-making. When poorly-calibrated, they can lead to predictable errors in judgment.[11] Unfortunately, heuristics do not respond to traditional educational efforts, which may explain the persistence of under-triage despite four decades of quality improvement efforts.[12]

In this study, we examined clinical records for evidence that physicians used one such heuristic (*representativeness*, or pattern recognition), specified its characteristics, and assessed the magnitude of its effect on triage decision-making. We hypothesized that physicians using this heuristic would be more likely to transfer patients who “fit” with their idea of severe injury compared to patients who did not, despite similar actual injury severity.

## Materials and methods

### Overview

We performed a retrospective cohort study of moderate-to-severely injured (Abbreviated Injury Scale, AIS ≥ 3) adult (age ≥ 18) patients evaluated initially at non-trauma centers in the University of Pittsburgh Medical Center (UPMC) system from January 2010 to December 2014. UPMC is an integrated health network in Western Pennsylvania, a region of approximately 4 million people, with over 30 hospitals and a 41% medical-surgical market share. Annually, UPMC hospitals process approximately 800,000 emergency department visits, and the UPMC Trauma Care System admits over 11,000 patients per year. The University of Pittsburgh Institutional Review Board approved the design and permitted a waiver of written consent given the retrospective nature of this study and minimal risk of harm to patients.

### Conceptual model and codebook development

Behavioral decision science research shows that people make judgments based on complex interactions between two cognitive systems. System 1 processes are fast, intuitive, and involve mental shortcuts (*heuristics*) relying on limited information. System 2 processes are slow, methodical, and employ deliberate analysis of all available data.[13] Under conditions of stress and time pressure, people instinctively default to the use of heuristics that can produce accurate answers quickly, but can also produce biased judgments.[14] One such heuristic is *representativeness*, defined by Kahneman and Tversky as the process of making judgments by determining “the degree to which an object/event is similar in essential characteristics to its parent population and to which it reflects the salient process by which it is generated.”[15] In other words, people judge the probability of an event based on how well a case fits a set’s archetype.[16]

Emergency physicians are trained to triage patients first by judging the severity of the injury and then determining the treatment options. We propose that they judge injury severity, in part, by using the representativeness heuristic. They search for case features that match their archetype of severe injury and are more likely to transfer patients who exhibit them. The ‘typical’ patient with moderate-to-severe injury may not manifest these representative characteristics and physicians would be less likely to transfer them. By extension, patients who exhibit these representative (archetypal) features may, in fact, be uncommon or the ‘atypical’ case.

In order to operationalize representativeness for severe trauma cases, we began by identifying a set of candidate features. We used the Center for Disease Control (CDC) Field Triage Guidelines 2011 and the ACS-COT Field Resources Guidelines as sources for potential cues.[1, 17] The most important themes in these guidelines were depressed level of consciousness, abnormal vital signs, anatomically-concerning injuries, and high-risk mechanisms. Using these themes as a guide, a multi-disciplinary group of regional and national emergency medicine, trauma surgery, and behavioral science experts (n = 7) produced a list of salient patient-level characteristics that might evoke the identification of a ‘representative’ severely-injured patient. Their task was to create a list of characteristics that could be easily coded from the electronic medical record, such that there was no ambiguity about their presence or absence. These characteristics included: abnormal level of consciousness (GCS < 14), hemodynamic instability, hypoxia, neurologic deficit, and open fracture. In addition to our list of patient-level characteristics, we identified a history of motor vehicle collision (MVC) or penetrating injury as being more representative of severe injury than other mechanisms like an assault or a fall.

We then examined five years of case records in the UPMC system, coding each record in terms of the presence or absence of each of those characteristics. We created a detailed codebook with definitions for each characteristic (see S1 Table). One coder (SK) reviewed all the charts. To assess inter-rater reliability, a second coder (DM) evaluated a random sample of 5% of the patient charts, with 91% total agreement (kappa = 0.82) for the various characteristics. We resolved disagreements between coders through consensus.

### Study population

All patients presenting to the UPMC system have data from their records abstracted into the institution’s Medical Archival System. By querying these archives, we identified adults (age ≥ 18) who presented to UPMC non-trauma centers between January 1, 2010 and December 31, 2014 with a primary discharge diagnosis of trauma (identified by ICD9-CM codes ranging from 800–959). We then mapped ICD9 codes to AIS scores to categorize patients with minor and moderate-to-severe injuries using a validated algorithm.[18] We classified patients with any injury diagnosis scored with an AIS ≥ 3 as having moderate-to-severe injury. For these patients, we extracted the evaluation and management (E&M) notes written by emergency physicians upon patients’ initial presentation from the Medical Archival System. Based on manual review of these notes, we excluded patients who had minor injuries that our algorithm inappropriately flagged as having AIS scores ≥ 3, patients who presented > 7 days after their initial injury, and patients who returned for follow-up after their initial hospital visit.

### Variables

We extracted patient age, sex, medical comorbidities, and discharge disposition from the medical records discharge abstract in the Medical Archival System. Using AIS scores, we calculated Injury Severity Scores (ISS) for each patient. Using ICD9 codes, we grouped diagnoses into categories of injuries that included traumatic brain injury (TBI), rib fracture, intraabdominal injury, spinal injury, pelvic fracture, and long bone fracture. Table 1 shows the map between our set of representative characteristics and these injury types for subgroup analysis.

**Table 1:** Representative characteristics and conceptual mapping to injuries.

	MVC^a^ or Penetrating Mechanism	Abnormal GCS^b^ (<14)	Neurologic Deficit	Hemodynamic instability	Hypoxia	Open fracture
Traumatic brain injury	X	X	X			
Rib fracture	X			X	X	
Intraabdominal injury	X			X		
Spinal injury	X		X			
Pelvic fracture	X			X		
Long bone fracture	X					X

^a^MVC = motor vehicle collision

^b^GCS = Glasgow Coma Scale

### Role of representativeness in decision-making

From the initial E&M notes, we abstracted evidence of mechanism of injury, the presence of representative characteristics, and the disposition decision (discharge home, admit to the non-trauma center, or contact a trauma surgeon for transfer to a trauma center). We reviewed the notes to determine how physicians made their disposition decision for the patient, and whether they explicitly noted any of the representative characteristics as influencing their decision. For patients who refused transfer or died in the emergency department while awaiting transfer, we credited the physician with intent to transfer.

### Statistical analyses

We summarized patient characteristics, injury types, and injury severity using means (standard deviations [SD]) for continuous data and proportions (%) for categorical data as appropriate. For each injury type, we performed bivariate analysis of the presence of expected representative characteristics and mechanism with intent to transfer using χ^2^ or Fisher’s Exact Test. We used a multivariable logistic regression model to estimate the association between representativeness and transfer and calculated adjusted odds ratios (aOR) and 95 percent confidence intervals (95% CI). In the model, we included patient age, sex, race (white or non-white), presence or absence of any representative characteristics, injury mechanism (fall/assault/crush, MVC, or penetrating), and ISS to adjust for differences in case mix. Random effects for hospital were used to account for clustering by institutional-level practice variations. To test for possible differences in the effect of representativeness on transfer by hospital, we included interaction terms between representativeness and hospital while continuing to adjust for the other covariates. We tested for significant heterogeneity in the effect of representativeness by hospital using I^2^.[19] This statistic ranges from 0–100% and estimates the percentage of variation in effect size due to heterogeneity across hospitals. We summarized the overall effect of representativeness across hospitals using the random effects method of DerSimonian and Laird, which incorporates weighted effects by hospital due to unbalanced clustering.[20] A p-value of < 0.05 was considered significant. All data were analyzed using Stata version 15 (StataCorp, TX).

## Results and discussion

### Patient characteristics

**Fig 1:**
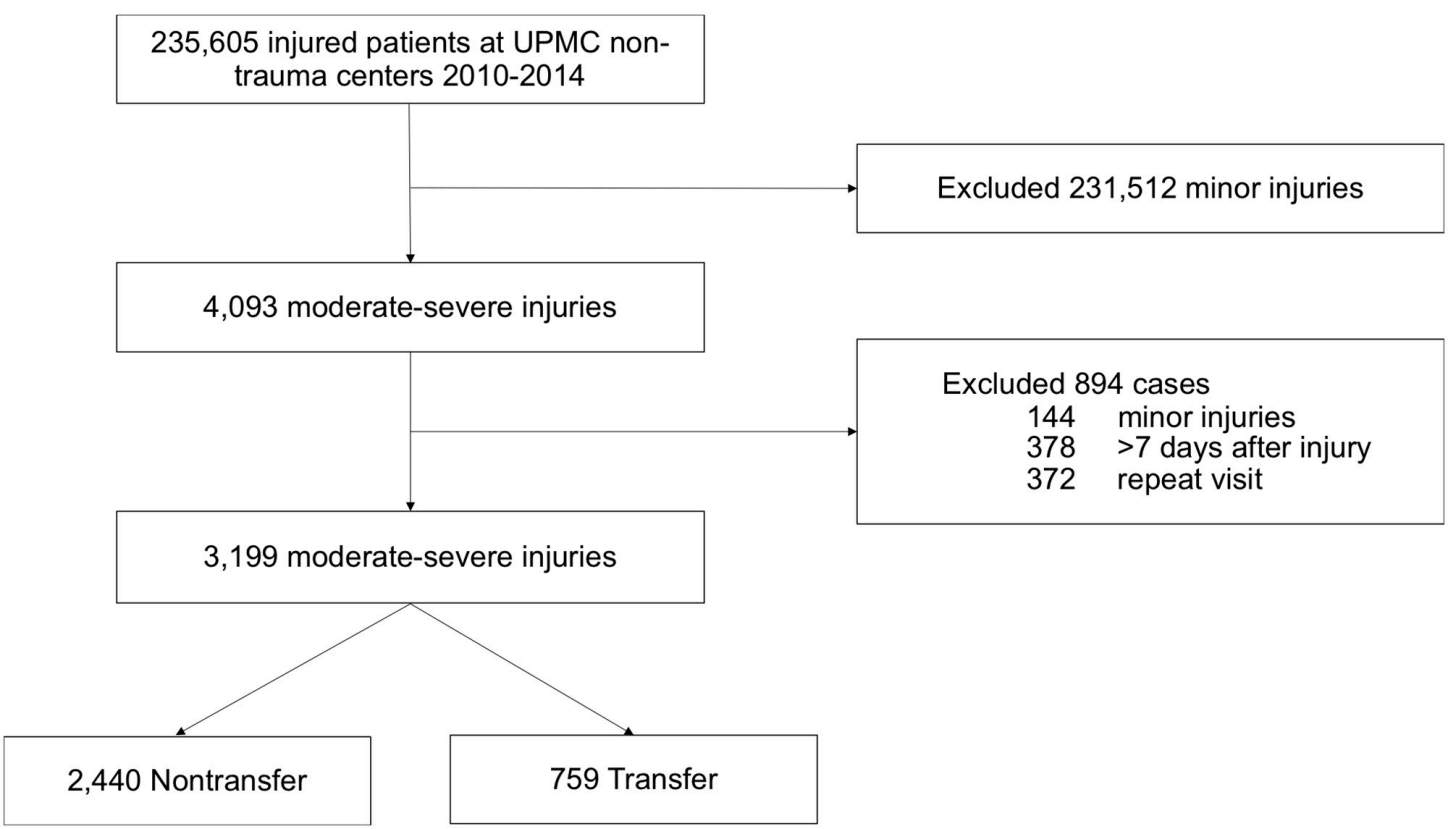
Cohort selection flow diagram.

We identified a total of 235,605 patient visits with a primary injury diagnosis who presented to nine UPMC non-trauma centers during the period of analysis. Of these, we initially identified 4,093 patients as having a moderate-to-severe injury. We excluded 894 cases after manual record review for actually having only minor injuries, presenting > 7 days after injury, or returning for treatment of a known injury. The remaining 3,199 (1%) cases with moderate-to-severe injuries served as our primary cohort (see Fig 1 and Table 2). The cohort had a median age of 78 years (SD 20.1), and mean ISS of 10.9 (SD 3.3). Physicians intended to transfer 759 (24%) of these patients. The majority of patients in the entire cohort had only one moderate-to-severe injury, most commonly pelvic fracture (28%) and long bone fracture (23%). 36 patients (1%) died prior to discharge from the hospital.

**Table 2:** Demographics and injury features (n = 3,199).

Variable	Value
Age, mean (SD)	71.2 (20.1)
Age ranges, n (%)	
< 70	1,188 (37)
70–79	511 (16)
80–89	1,014 (32)
≥ 90	486 (15)
Sex, n (%)	
Male	1,217 (38)
Female	1,982 (62)
ISS^a^, mean (SD)	10.9 (3.5)
< 15	2,759 (86)
> 15	440 (14)
Comorbidities, n (%)	
Chronic obstructive pulmonary disease	596 (19)
Congestive heart failure	388 (12)
Hypertension	1,740 (54)
Kidney disease	240 (8)
Diabetes	619 (19)
Cirrhosis	47 (1)
Anemia	431 (13)
Mechanism, n (%)	
Assault	132 (4)
Crush	115 (3)
Fall	2,740 (86)
MVC^b^	178 (6)
Penetrating	34 (1)
AIS regions ≥ 3, n (%)	
Head	819 (26)
Face	0
Chest	813 (25)
Abdomen	85 (3)
Extremity	1,535 (48)
External	1 (0)
No. of regions with AIS^c^ ≥ 2, n (%)	
1	2, 643 (83)
2	508 (16)
3	47 (1)
4	1 (0)
Specific Injuries n, (%)	
Traumatic brain injury	679 (21)
Rib fracture	683 (21)
Intraabdominal injury	71 (2)
Spinal injury	152 (5)
Pelvic fracture	883 (28)
Long bone fracture	747 (23)

^a^ISS = Injury Severity Score

^b^MVC = motor vehicle collision

^c^AIS = Abbreviated Injury Scale

Patients who were not transferred were generally older than those who were transferred (median 73 years, SD 19.0 vs. median 65 years, SD 21.8) and more likely to be female (66% vs. 48%). Non-transferred patients were also more likely to be comorbid, with higher rates of chronic obstructive pulmonary disease (20% vs. 14%), congestive heart failure (14% vs. 8%), hypertension (57% vs. 45%), chronic kidney disease (9% vs. 2%), and anemia (15% vs. 6%).

### Role of representativeness in decision-making

Chart review of E&M notes provided qualitative evidence of the representativeness heuristic in triage. For example, one provider noted:

“Chest x-ray interpreted by me showed multiple left-sided rib fractures with some possible left-sided effusion. Because of the patient's hypoxia as well as her multiple rib fractures as well as the mechanism of injury [MVC], I discussed the case with Dr.** at [trauma center] who recommended the patient be transferred to [trauma center] as a level 2 trauma.”

In contrast, we also found examples where the failure to recognize the salience of injury characteristics played a role in decision-making. In one instance, a different provider stated:

“CT of his chest and abdomen showed nondisplaced fractures of the 4th through the 10th left ribs and also a small pneumothorax and also mild pulmonary contusion. I spoke with Dr. **, a pulmonologist. He looked at the CT scan and decided the patient should be admitted to the hospitalist to a telemetry bed.”

Additional qualitative examples of the influence of representativeness on decision-making can be found in Table 3.

**Table 3:** Medical decision-making of representative and Non-representative injuries.

Traumatic brain injury	
Representative	“Patient lethargic. Does not follow commands. Very little verbalization. Subdural hematoma on CT scan. I did call for transfer. I spoke to Dr. ** who has accepted the patient.”
Non-representative	“This is a 90-year-old female who was found to have atrial fibrillation on EKG as well as a left frontal intraparenchymal hemorrhage on head CT with subarachnoid and subdural hemorrhage. The patient was also found to have an occipital skull fracture. The patient was found to have a troponin of 0.7. The patient remained completely asymptomatic throughout her Emergency Department course. She was admitted for further evaluation and management of her intracranial bleeding and elevated troponins.”
Rib fracture	
Representative	“Chest x-ray interpreted by me showed multiple left-sided rib fractures with some possible left-sided effusion. Because of the patient's hypoxia as well as her multiple rib fractures as well as the mechanism of injury [MVC], I discussed the case with Dr.** at [trauma center] who recommended the patient be transferred to [trauma center] as a level 2 trauma.”
Non-representative	“CT of his chest and abdomen showed nondisplaced fractures of the 4th through the 10th left ribs and also a small pneumothorax and also mild pulmonary contusion. I spoke with Dr. **, a pulmonologist. He looked at the CT scan and decided the patient should be admitted to the hospitalist to a telemetry bed.”
Intraabdominal injury	
Representative	“Impression was that there was irregular appearance of the spleen on noncontrast images with hemoperitoneum, consistent with partial splenic rupture. I hesitate to give him additional pain medication at this time, due to his blood pressure being on the lower side. Beyond that, the patient after finding the initial splenic laceration and hemoperitoneum, had immediate consultation through Med Call Trauma Surgeon, Dr.**. She has agreed to accept the patient for transfer.”
Non-representative	“She has evidence of gross hematuria and what I suspect is a perinephritic versus intrarenal hematoma. I discussed the case with Dr. Woodburn who agreed to accept the patient for admission. Consult to Dr. ** of Urology. Admitted in stable condition.”
Pelvic fracture	
Representative	“Left inferior pubic rami fracture. Widening of the symphysis pubis. Acetabular disruption. His heart rate is slightly elevated. Given the severity of his injury, the mechanism of injury [MVC], and that he is anticoagulated he is better served at a trauma center. I've spoken to Dr. **, who accepted the patient in transfer.”
Non-representative	"This is a 62-year-old female who presents emergency room after fall from a ladder and sustained an unstable pelvis fracture. She is hemodynamically stable. Patient is being admitted to orthopedic floor under hospitalist service.”
Spinal injury	
Representative	“Based on the new C2 fracture as well as some left arm tingling, we feel it is best the patient be transferred to [trauma center] for evaluation by the trauma team and the neurosurgeons.”
Non-representative	“I discussed the case with the orthopedic resident to evaluate the patient. It was felt that the patient only needed a soft cervical collar and did not need to be admitted for the nondisplaced odontoid fracture.”
Long bone fracture	
Representative	“19-year-old male with a gunshot wound to the left lower extremity that has shattered the fibula. Discussed with Dr. **, Trauma surgeon at [trauma center], presently and he accepts him for transfer.”
Non-representative	“71-year-old female with right proximal humerus fracture and right femoral neck fracture. Discussed with patient and patient’s family, eventually to be admitted with orthopedic consultation.”

### Outcomes

Representative characteristics occurred in 719 patients (22%) of the cohort. Among these patients, 623 (87%) had one characteristic, 68 (9%) had two, 23 (3%) had three, and five (1%) had four. ISS was correlated with the number of representative characteristics. Patients without any representative characteristics had a mean ISS of 10.8 (SD 3.1), while those with four characteristics had a mean ISS of 21.6 (SD 7.1). Nonetheless, of the patients with ISS > 15, 311 (71%) had no representative characteristics. The presence of representative characteristics was associated with an increased likelihood of being transferred. Overall, 224 (30%) transferred patients had at least one representative characteristic, compared to 495 (20%) of patients who were not transferred (p < 0.001). Analysis of representative characteristics by injury subgroup showed similar findings. For example, open fracture was significantly more common (29%) in transferred patients with long bone fractures than in those who were not transferred (10%). In addition, transferred patients with any injury were more likely to have been in an MVC or had a penetrating injury as opposed to a fall or other mechanism. Table 4 shows the association of representative characteristics and mechanisms with transfer decisions by injury subgroup.

**Table 4:** Transfer decisions by injury and representativeness.

Variable	Nontransfer	Transfer	p-value
Traumatic brain injury, n (%)	n = 373	n = 306	
No representative characteristics	345 (93)	270 (88)	0.13
Abnormal GCS^a^	7 (2)	13 (4)	
Neurologic deficit	16 (4)	14 (5)	
Both representative characteristics	5 (1)	9 (3)	
Mechanism			0.11
Assault	46 (12)	26 (9)	
Crush	1 (0)	2 (1)	
Fall	315 (84)	261 (85)	
MVC^b^	11 (3)	15 (5)	
Penetrating	0	2 (1)	
Rib fracture, n (%)	n = 513	n = 170	
No representative characteristics	415 (81)	119 (70)	0.001
Hypoxia	37 (7)	20 (12)	
Hemodynamic instability	50 (10)	18 (10)	
Both representative characteristics	11 (2)	13 (8)	
Mechanism			< 0.001
Assault	12 (2)	12 (7)	
Crush	1 (0)	2 (1)	
Fall	469 (92)	119 (70)	
MVC	30 (6)	30 (18)	
Penetrating	1 (0)	7 (4)	
Intraabdominal injury, n (%)	n = 37	n = 34	
No representative characteristics	33 (89)	23 (68)	0.04
Hemodynamic instability	4 (11)	11 (32)	
Mechanism			< 0.001
Assault	1 (3)	5 (15)	
Crush	0	0	
Fall	33 (89)	13 (38)	
MVC	2 (5)	11 (32)	
Penetrating	1 (3)	5 (15)	
Spinal injury, n (%)	n = 78	n = 74	
No representative characteristics	72 (92)	67 (91)	0.78
Neurologic deficit	6 (8)	7 (9)	
Mechanism			0.04
Assault	0	1 (1)	
Crush	0	1 (1)	
Fall	75 (96)	63 (86)	
MVC	3 (4)	9 (12)	
Penetrating	0	0	
Pelvic fracture, n (%)	n = 842	n = 41	
No representative characteristics	749 (89)	37 (90)	> 0.99
Hemodynamic instability	93 (11)	4 (10)	
Mechanism			< 0.001
Assault	3 (0)	0	
Crush	0	0	
Fall	822 (98)	30 (73)	
MVC	17 (2)	11 (27)	
Penetrating	0	0	
Long bone fracture, n (%)	631	116	
No representative characteristics	569 (90)	82 (71)	< 0.001
Open fracture	62 (10)	34 (29)	
Mechanism			< 0.001
Assault	9 (1)	4 (3)	
Crush	92 (15)	12 (10)	
Fall	510 (81)	84 (73)	
MVC	13 (2)	11 (10)	
Penetrating	7 (1)	5 (4)	
GCS = Glasgow Coma Scale; MVC = motor vehicle collision			

^a^GCS = Glasgow Coma Scale

^b^MVC = motor vehicle collision

**Fig 2:**
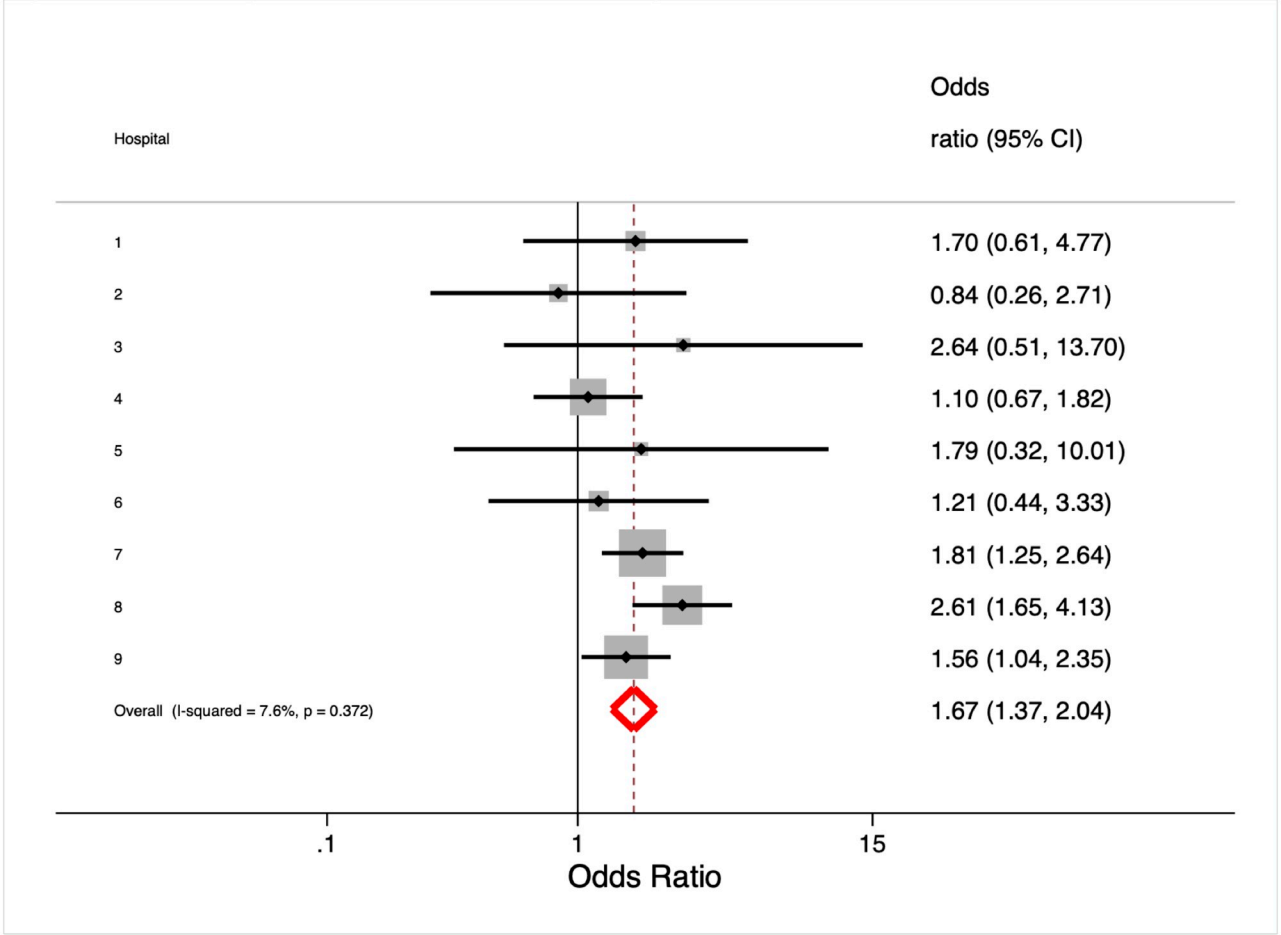
Effects of representativeness on transfer by hospital. Boxes indicate effect of representiveness on transfer for each hospital. Box size is inversely proprotinal to the variance of each hospital effect. Lines indicate 95% confidence intervals. Diamond indicates random effects summary across hospitals.

After adjusting for age, sex, race, and injury severity, the presence of any representative characteristics was associated with higher odds of transfer (aOR 1.7, 95% CI: 1.4–2.0, p < 0.001). The individual hospital-level effects of representativeness showed some variation, but the test of heterogeneity of this effect was not statistically significant (p = 0.37), and the I^2^ was low (8%). These are shown in Fig 2. We also found that the odds of transfer were higher with MVCs (adjusted OR 2.4, 95% CI: 1.7–3.3, p < 0.001) and penetrating injuries (adjusted OR 3.8, 95% CI: 1.7–8.7, p = 0.001), two representative mechanisms. Finally, men were more likely to be transferred than women (adjusted OR 1.7, 95% CI: 1.4–2.0, p < 0.001).

### Principal findings

Our findings help address one potential cause of under-triage at non-trauma centers—the use of the representativeness heuristic. Patients with moderate-to-severe injury present uncommonly, and they seldom manifest signs that physicians might consider to be ‘representative’ of severe injury. As a result, physicians at non-trauma centers may lack the opportunity to calibrate their heuristics to national triage guidelines, leading to predictable under-triage of moderate-to-severely injured patients. Understanding the nature of this cognitive bias can aid the design of behavioral interventions to improve triage decision-making.

Our results help validate the finding that the representativeness of a case reflects an associative process informed by experience and training.[10–12] In order for people to develop intuitive expertise, they must function within a decisional context in which the cues to the problem are valid. They also require opportunities to learn relevant cues and recalibrate their decisions based on outcome feedback.[21] Physicians using the representativeness heuristic may fail to recognize and appropriately transfer patients with moderate-to-severe trauma due to the low overall incidence of such injuries and the even lower prevalence of representative characteristics. Other research confirms that physician discrimination of moderate-to-severe injuries from minor injuries improves with greater caseload.[22] This likely represents improved heuristic calibration with practice and feedback. However, real-world injury patterns do not provide emergency physicians in the community enough feedback to develop a well-calibrated associative process.

In light of decision-making difficulties stemming from the low caseload of moderate-to-severe trauma, the temptation may be to remedy under-triage by prescriptively lowering transfer thresholds. However, changing the decision rule without improving understanding can result in significant over-triage, so that patients with minor injuries consume and potentially overwhelm trauma center resources. Although the ACS-COT advocates disproportionate over-triage (up to 50%) to avoid under-triage (goal less than 5%), we have previously shown that compliance with these guidelines would result in a five-fold increase in trauma center transfers. For example, in Pennsylvania where there are roughly 3,600 transfers per year, following these guidelines would mean about 15,000 more transfers to trauma centers annually.[6] Morbidity and mortality can increase as hospital resources and staff are overwhelmed.[23, 24] Simply increasing trauma center patient volume without reallocating resources would overburden hospitals and likely result in worse care. Instead, one strategy to improve under-triage is to capitalize on our knowledge of heuristics to elicit behavioral modifications. These can improve injury discrimination and clinical judgment without drastically changing decision rules.[25] Better calibration of physician heuristics might increase the transfer of patients most benefiting from trauma center services and ensure the best use of health system resources.

Institutional norms can also influence decision-making if hospitals exert implicit or explicit pressure on emergency physicians to retain patients in-house. Prior work has shown that geography, hospital profit status, and patient payor source can be powerful driving factors in failure to transfer.[7] Our analyses found that representativeness played a consistent role across our hospitals, lending credence to the prominence of the representativeness heuristic in triage decisions. We also found that younger patients and female patients were more likely to be transferred, consistent with prior observations of disparities in trauma triage.[26–28] Perhaps these characteristics influence the representativeness of a case (i.e. younger men demonstrate more signs ‘typical’ of severe injury than older women). Some types of injuries were also more likely to be under-triaged. Our qualitative analysis suggests that these injuries not only lacked features representative of severe injury, but they might have prompted thinking in the opposite direction. Physicians frequently referred to the consultants and resources at their hospital when making plans to manage the patients’ injuries, suggesting that under-triage might have also occur because they over-estimated the abilities of their facilities. They may have simply been seeking advice or attempting to avoid over-triage to trauma centers. But sometimes, they appeared to prefer making referrals to colleagues with whom they had a relationship instead of sending patients to anonymous trauma surgeons at a tertiary care center, suggesting that local physician networks may influence triage practices. These observations provide additional possible loci for intervention and warrant further evaluation.

This study is limited by our reliance on administrative data which required us to make inferences about injury identification and injury severity discrimination. We sought to minimize that bias by manually reviewing the records of all patients identified by our initial screening in order to confirm their suitability and injury severity estimates. A second limitation is that we developed our set of representative characteristics deductively instead of inductively, relying on existing guidelines and expert opinion. We focused on a robust core of characteristics and mechanisms that were most salient for decision-making. Doing so allowed for reliable coding of cases, but we likely missed some characteristics. A more inclusive set of characteristics would likely enhance rather than reduce the magnitude of the effect of representativeness on transfer. Third, our study sample included a large proportion of geriatric patients and may lack generalizability. However, the elderly constitute a substantial portion of trauma-related admissions, in-hospital deaths, and under-triage.[29–30] As the population ages substantially over the next several decades, geriatric trauma management will become increasingly important.[31] Our standard for appropriate transfer decisions was the ACS triage guidelines, which represent the standard of care in the U.S. and have been endorsed by major stakeholders. They mandate transfer of moderate-to-severely injured patients including those with femur fractures, open extremity fractures, and pelvic fractures. Deviation from these guidelines may or may not be warranted, but regardless, can allow for inferences into the cognitive processes that drive decision-making. And finally, we acknowledge that physician training and risk preference also drive decision-making. In this study, we chose to focus on heuristics as one potential locus of variation.

### Conclusions

We found that moderate-to-severely injured patients often do not have features representative of severe injury, and this impacts triage decisions by emergency physicians. This suggests that recalibrating the heuristics that physicians use to identify severely-injured patients might reduce predictable under-triage and improve outcomes after trauma.

## Supporting information

S1 Table Evaluation and management note coding definitions. (PDF)
