# Supplementary material for: Defining the representativeness heuristic in trauma triage: A retrospective observational cohort study
Source: PLoS One. 2019 Feb 8;14(2):e0212201. doi: 10.1371/journal.pone.0212201 (PMC6368323; doi:10.1371/journal.pone.0212201)
Supplement: S1 Table — (PDF) [file pone.0212201.s001.pdf]

S1 Table. Evaluation and Management Note Coding Definitions[a]

Mechanism	• Categorized as motor vehicle collision, penetrating injury, fall, assault, crush
Abnormal GCS	• Glasgow Coma Scale (calculated from documented physical exam) < 14
Neurological deficit	• Lateralizing signs (unequal pupils, motor or sensory deficit)
Hemodynamic instability	• Heart rate > 100 beats per minute<br>• Systolic blood pressure < 90 or > 180 mmHg
Hypoxia	• Oxygen saturation < 92%<br>• Intubation<br>• Chest wall crepitus<br>• Deterioration from baseline oxygen requirement
Open fracture	• Obvious open fracture<br>• Fracture with overlying wounds
Disposition	• Categorized as admission, discharge home, or transfer<br>• Transfer definitions: actual transfer to trauma center, documentation of intent to transfer but patient/family refused, admission for comfort measures only

[a]Characteristics were coded as present when definitions were explicitly documented, otherwise they were coded as absent
